# Intracerebral electrographic activity following a single dose of diazepam nasal spray: A pilot study

**DOI:** 10.1002/epi4.12890

**Published:** 2024-01-04

**Authors:** Michael D. Privitera, Lucy C. Mendoza, Enrique Carrazana, Adrian L. Rabinowicz

**Affiliations:** ^1^ Department of Neurology University of Cincinnati College of Medicine Cincinnati Ohio USA; ^2^ Neurelis, Inc. San Diego California USA; ^3^ John A. Burns School of Medicine University of Hawaii Honolulu Hawaii USA

**Keywords:** acute repetitive seizure, epilepsy, epileptiform activity, rescue medication, responsive neurostimulation, seizure cluster

## Abstract

**Objective:**

Rescue benzodiazepine medication can be used to treat seizure clusters, which are intermittent, stereotypic episodes of frequent seizure activity that are distinct from a patient's usual seizure pattern. The NeuroPace RNS® System is a device that detects abnormal electrographic activity through intracranial electrodes and administers electrical stimulation to control seizures. Reductions in electrographic activity over days to weeks have been associated with the longer‐term efficacy of daily antiseizure medications (ASMs). In this pilot study, electrographic activity over hours to days was examined to assess the impact of a single dose of a proven rescue therapy (diazepam nasal spray) with a rapid onset of action.

**Methods:**

Adult volunteers (>18 years old) with clinically indicated RNS (stable settings and ASM usage) received a weight‐based dose of diazepam nasal spray in the absence of a clinical seizure. Descriptive statistics for a number of detections and a sum of durations of detections at 10‐min, hourly, and 24‐h intervals during the 7‐day (predose) baseline period were calculated. Post‐dose detections at each time interval were compared with the respective baseline‐detection intervals using a 1 SD threshold. The number of long episodes that occurred after dosing also were compared with the baseline.

**Results:**

Five participants were enrolled, and four completed the study; the excluded participant had recurrent seizures during the study. There were no consistent changes (difference >1 SD) in detections between post‐dose and mean baseline values. Although variability was high (1 SD was often near or exceeded the mean), three participants showed possible trends for reductions in one or more electrographic variables following treatment.

**Significance:**

RNS‐assessed electrographic detections and durations were not shown to be sensitive measures of short‐term effects associated with a single dose of rescue medication in this small group of participants. The variability of detections may have masked a measurable drug effect.

**Plain Language Summary:**

Rescue drugs are used to treat seizure clusters. Responsive neurostimulation (RNS) devices detect and record epilepsy brain waves and then send a pulse to help stop seizures. This pilot study looked at whether one dose of a rescue treatment changes brain activity detected by RNS. There was a very wide range of detections, which made it difficult to see if or how the drug changed brain activity. New studies should look at other types of brain activity, multiple doses, and larger patient groups.

**Key Points**

- There is a need to identify new end points that could better characterize the effects of rescue medications used for seizure clusters.
- Electrographic activity measured with responsive neurostimulation (RNS) has been associated with the efficacy of daily antiseizure drugs.
- In this hypothesis‐testing, pilot study, detections were analyzed in participants with RNS who received 1 dose of diazepam nasal spray interictally.
- Day‐to‐day and hour‐to‐hour electrographic activity was highly variable within participants, and data were unsuitable for statistical analysis.
- Future studies could consider other electrographic variables, participant selection criteria, multiple dosing, and cycles of epilepsy.

## 1 INTRODUCTION

Seizure clusters are intermittent, stereotypic episodes of frequent seizure activity that are distinct from a patient's usual seizure pattern.^1^, ^2^, ^3^ Seizure clusters vary from patient to patient and can persist for 24 h or more.^4^, ^5^ Diazepam rectal gel, midazolam nasal spray, and diazepam nasal spray are approved by the US Food and Drug Administration for rescue treatment of seizure clusters.^1^, ^2^, ^3^ Rescue medications are typically evaluated on their ability to control seizure clusters for a specified duration (e.g., 6, 12, 24 h).^6^, ^7^, ^8^, ^9^, ^10^ Examples of outcomes related to seizure‐cluster control include seizure frequency/count, recurrence, or time to termination.^6^, ^8^, ^9^ Patient usage of second doses and observation of time between clusters also have been used as proxy measures for effectiveness.^10^, ^11^

The RNS® System (NeuroPace, Inc.) is a device that uses intracranial electrodes to detect abnormal electrographic activity (seizures, counts and durations of detections, prolonged epileptiform activity [long episodes]) as well as administer electrical stimulation (responsive neurostimulation [RNS]) to attenuate seizure activity over time.^12^, ^13^ RNS is an objective, real‐time measure of electrographic activity that has been used to predict the long‐term efficacy of antiseizure medications (ASMs).^14^, ^15^, ^16^ However, electrographic activity captured by RNS has not been used to assess the efficacy of rescue medications, which are intermittently used for the acute treatment of seizure clusters. Therefore, the purpose of this pilot study was to analyze detections of electrographic activity over short durations (minutes–hours) following a single dose of diazepam nasal spray, which is indicated for the acute treatment of intermittent, stereotypic episodes of frequent seizure activity (i.e., seizure clusters, acute repetitive seizures) that are distinct from a patient's usual seizure pattern in patients with epilepsy aged 6 years and older.^1^

## 2 METHODS

### 2.1 Study design

This was a pilot study to examine the effects of a single dose of diazepam nasal spray on electrographic activity as measured with RNS. The study was conducted at the Epilepsy Center of the University of Cincinnati Gardner Neuroscience Institute. The protocol was approved by the Institutional Review Board, and all participants provided written informed consent. The study adhered to ethical principles originating from the Declaration of Helsinki and was consistent with Good Clinical Practice and applicable regulatory requirements.

The study design consisted of baseline, open‐label treatment, and follow‐up periods. Electrocorticographic data were collected per routine clinical care with the RNS system and uploaded daily to the Patient Data Management System 7 days before (baseline period) and 7 days following the dose of diazepam nasal spray (follow‐up period). Assessment for RNS inclusion/exclusion criteria was conducted during the baseline period, and data were used for comparisons to post‐dose periods. The treatment period consisted of a single, weight‐based dose of diazepam nasal spray administered in the clinic between 10:00 a.m. and 12:20 p.m. (non‐ictal state), followed by 4 h of observation in the clinic. Intermittent electrocorticograms, vital signs, Columbia Suicide Severity Rating Scale, and Stanford Sleepiness Scale were collected during the observation period. Participants who scored a 4 or higher on the Stanford Sleepiness Scale were evaluated for discharge from the 4‐h observation period.

### 2.2 Participants

Men or women >18 years of age and weighing >50 kg, with focal epilepsy consistent with the International League Against Epilepsy (ILAE) criteria supported by either electroencephalography or magnetic resonance imaging data and meeting the ILAE definition of refractory epilepsy were eligible for inclusion. Participants had RNS implanted for at least 3 months as part of their usual clinical care, with detection and stimulation settings stable for at least 30 days before and during the study. Participants needed to have no change in dosages of ASMs or vagus nerve stimulation settings 30 days before and during the study and have a minimum mean rate of detections of 5 per hour with no fewer than 2 detections in any hour between 9:00 a.m. and 5:00 p.m. during the baseline period. Exclusion criteria consisted of the following: pregnancy; history of substance abuse within the previous 2 years; unable to provide consent; use of recreational or medicinal marijuana, cannabinoids, and/or derivatives other than stable dosing of oral‐solution cannabidiol (Epidiolex®); current chronic opioid use; history of poor medication compliance as judged by the investigator; any medical or psychiatric condition that the investigator believed would impair reliable participation in the trial; participating or previous participation in an investigational product/device trial in the preceding 30 days before study entry; diagnosis of psychogenic or nonepileptic seizures; use of rescue benzodiazepines <2 weeks before baseline‐detection rate assessment (stable doses of a prescribed benzodiazepine for 30 days before study entry were permitted); and having a clinical seizure 96 h before or 48 h following administration of diazepam nasal spray.

### 2.3 Safety

Adverse events not present at baseline or those that were exacerbated with treatment (treatment‐emergent adverse events [TEAEs]) were collected. TEAEs were assessed for 7 days after administration of diazepam nasal spray. Tolerability assessments included the Stanford Sleepiness Scale and vital signs taken during the in‐clinic observation period.

### 2.4 Data analysis

**FIGURE 1:**
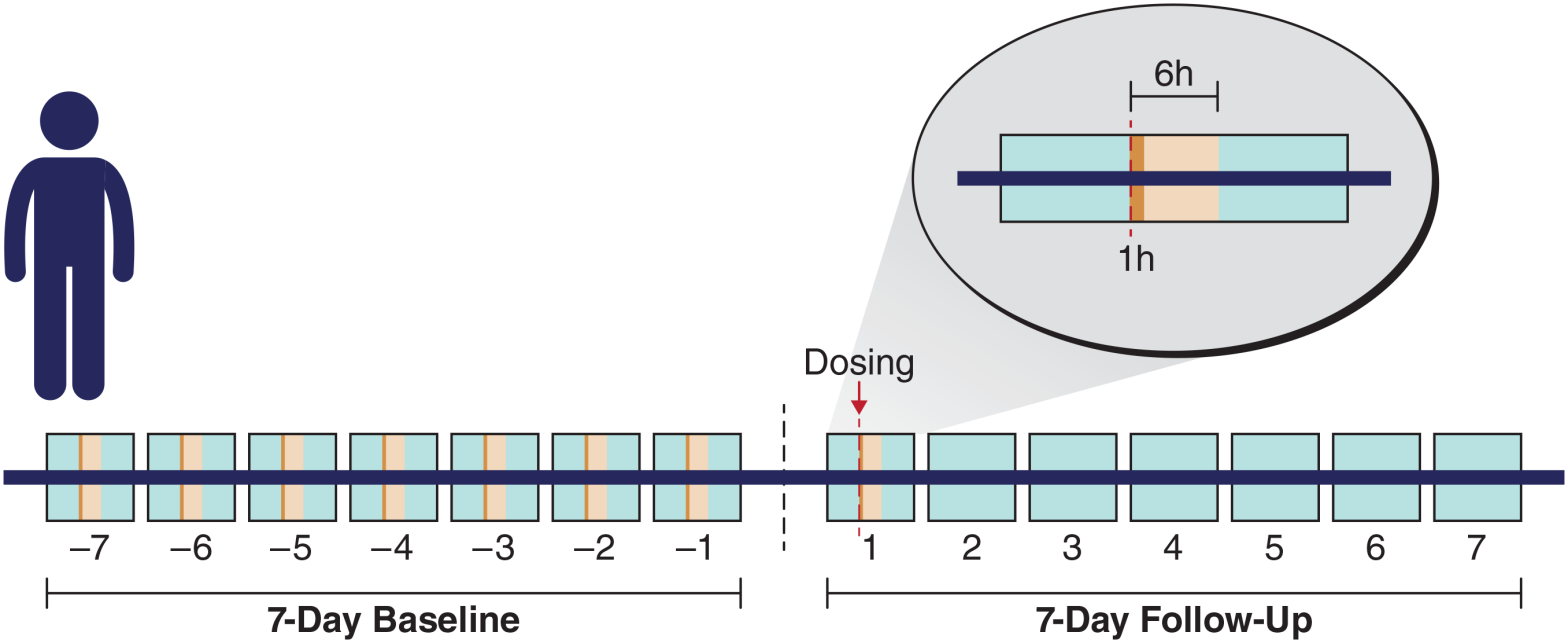
Analysis of electrographic activity. Electrographic activity (number and sum of durations of detections) at 10‐min, hourly, and 24‐h intervals post‐dose were compared to their respective 7‐day mean (SD) baseline values. For purposes of illustration, a single 1‐h interval is shown post‐dose, which was compared to the 7‐day mean baseline value for that same hour; all twenty‐four 1‐h intervals post‐dose were compared to their respective 7‐day mean baseline values for each hour. The 24‐h intervals post‐dose were compared to the 7‐day mean baseline value. The 10‐min intervals were collected during the 6 h after dosing; thirty‐six 10‐min intervals post‐dose were compared to their respective 7‐day mean baseline values for each 10‐min interval.

NeuroPace provided time‐marked detection counts, detection durations, and long episodes generated by the RNS system for each participant during the pre‐ and post‐dose periods. Descriptive statistics for this pilot study included the number of detections and the sum of durations of detections during the 7‐day baseline period (mean, SD). Detections at different time intervals post‐dose were compared with the respective means (SDs) at baseline (Figure 1). First, the number and sum of durations of detections during each hour of the 24‐h period post‐dose were compared with the mean 7‐day baseline values for that same hour (e.g., detections between 2:00 p.m. and 3:00 p.m. post‐dose were compared with mean 7‐day baseline detections between 2:00 p.m. and 3:00 p.m.). Second, the number and sum of durations of detections during each 10‐min interval of the 6‐h period post‐dose were compared with the mean 7‐day baseline values for that same 10‐min interval and hour (e.g., if the participant was dosed at 10:30 a.m., detections between 10:30 a.m. and 10:39 a.m. post‐dose were compared with mean 7‐day baseline detections between 10:30 a.m. and 10:39 a.m.). Third, the number and sum of durations over each 24‐h period for the duration of the follow‐up period (7 days post‐dose) were compared with the mean 7‐day baseline value for the 24‐h period. The number of long episodes recorded during the 7‐day period post‐dose was compared with the number of long episodes during the 7 days before dosing. Meaningful differences in post‐dose values were defined as a difference of >1 SD from the mean baseline value for this pilot study (e.g., comparing the number of detections from 11:00 a.m. to 12:00 p.m. post‐dose vs the 7‐day baseline mean number of detections from 11:00 a.m. to 12:00 p.m.).

## 3 RESULTS

Five participants (mean [SD] age: 32.0 [7.3] years, 60% female) met inclusion criteria and provided informed consent (Table 1). Participant 2 experienced a seizure during the 4‐h observation period and had clinical seizures during an additional baseline observation period; this participant was excluded from the analysis. Participant 1 fell asleep 2 h after dosing because of self‐reported sleep deprivation, constituting the only TEAE of the study, and was re‐dosed at another date. However, technical issues with data collection (missing 10‐min data) during the second dosing of participant 1 precluded full analysis; therefore, data from the initial dosing were used. Notably, this participant displayed substantially different responses to treatment at hourly time points, with the initial dose showing a trend for lower detections at the 24‐h interval while the re‐dosing showed no trend. Participant 4 had high electrographic activity during menses (detection counts were up to 10 times higher at the onset of menses compared to post‐menses); therefore, dosing was conducted between menses. No dosage or technical issues were reported for participants 3 and 5. All participants tolerated the diazepam nasal spray. There were no clinically relevant changes observed in vital signs or the Columbia Suicide Severity Rating Scale. Other than the participant who fell asleep, Stanford Sleepiness Scale scores were unaffected for 90 min, and all participants maintained a rating of 1 (feeling active, vital, alert, or wide awake) or 2 (functioning at high levels, but not at peak; able to concentrate) throughout the 4‐h observation period.

**TABLE 1:** Participant characteristics.

Participant	Sex	Age, y	Date of RNS implant	Electrode placement
1	Female	30	8/19/2019	Bitemporal
2^a^	Male	35	1/25/2021	Frontal
3	Female	21	1/9/2019	Bitemporal
4	Female	33	6/12/2017	Bitemporal
5	Male	41	2/20/2017	Unilateral temporal

Abbreviation: RNS, responsive neurostimulation.

^a^ Participant had seizure during visit and was excluded from the analysis.

**FIGURE 2:**
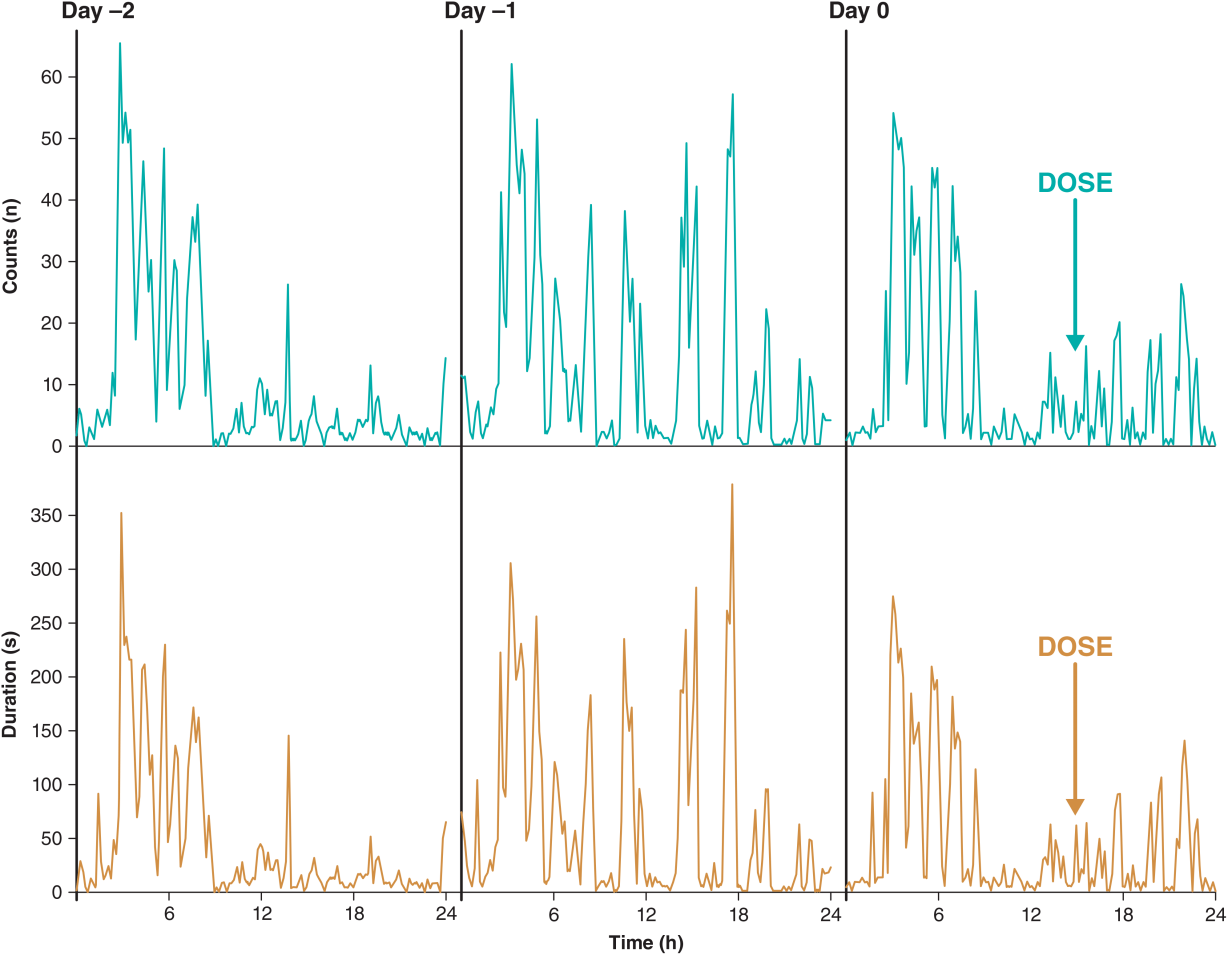
Representative responsive neurostimulation detections and sums of durations of detections.

The proportions of detections (number, duration) that were 1 SD lower than their respective mean baseline values are presented in Table 2. There were no consistent changes in detections using the 1 SD threshold to define a meaningful difference between post‐dose and mean baseline values. Variability (SD) in detections and the durations of detections was high for all participants during the baseline period for 10‐min and 1‐h intervals, with the SD similar to or more than the mean (Figure 2). The variability of these measures during the baseline 24‐h interval was high for 3 participants. Participant 5 had relatively low 24‐h‐interval variability in baseline detections and durations of detections, and a meaningful reduction in these measures was recorded post‐dose (Table 2). Overall, there were no apparent differences in results when comparing changes in the number of detections vs the sum of durations of detections; high amounts of baseline fluctuations at both 10‐min and 1‐h intervals were observed (Figure 2). Long episodes were infrequent during the 48‐h period post‐dose and showed no consistent pattern comparing counts baseline vs post‐dose (data not shown). The short duration of electrocorticography recordings done during the in‐person visit precluded meaningful analysis.

**TABLE 2:** Detection counts and durations.^a^

Participant	10‐min		1‐h		24‐h	
	Count *n*/*N* (%)	Duration *n*/*N* (%)	Count *n*/*N* (%)	Duration *n*/*N* (%)	Count *n*/*N* (%)	Duration *n*/*N* (%)
1^b^	13/36 (36.1)	14/36 (38.9)	12/24 (50.0)	12/24 (50.0)	4/6 (66.7)	4/6 (66.7)
3	14/36 (38.9)	13/36 (36.1)	6/24 (25.0)	7/24 (29.2)	2/7 (28.6)	2/7 (28.6)
4	14/36 (38.9)	10/36 (27.8)	7/24 (29.2)	6/24 (25.0)	1/5 (20.0)	1/5 (20.0)
5	7/36 (19.4)	8/36 (22.2)	10/24 (41.7)	9/24 (37.5)	7/7 (100)	7/7 (100)

^a^ Number (proportion) of intervals during observation >1 SD below the respective baseline mean value.

^b^ These data are from the initial dosing for participant 1. Data from re‐dosing were incomplete because of an error in downloading data. All other participants had only 1 dosing session.

## 4 DISCUSSION

The seizure diary is the gold standard for tracking clinical seizures, and information from seizure diaries is frequently used in clinical trials.^13^ However, a shortcoming of seizure diaries is that some seizures may not be recognized and recorded,^17^ especially if patients are alone at the time of seizure or if the seizure occurs during sleep. In the context of clinical trials of ASMs, the use of electrographic activity via RNS represents, perhaps, a more sensitive method to evaluate the efficacy of ASMs through their ability to detect subtle, subclinical events.^13^ Although electrographic activity from RNS has been used to characterize the efficacy of daily ASMs,^14^, ^15^, ^16^ the potential for RNS to detect changes in electrographic activity associated with intermittently used rescue medication was unclear. In this pilot study, we examined electrographic variables of counts and durations of detections as assessed with RNS in participants before and immediately following the administration of rescue medication, a single dose of diazepam nasal spray. Overall, there appeared to be trends for reductions in both of these electrographic variables at 10‐min, 1‐h, and 24‐h intervals post‐dose; however, the magnitude of variability in baseline data was substantial, with SDs near or exceeding the mean baseline values.

The long‐term efficacy of ASMs used as daily treatment has been associated with reductions in electrographic activity obtained with RNS,^14^, ^15^, ^16^ which involves ambulatory monitoring that minimizes potentially confounding factors, such as those described in epilepsy monitoring unit studies (e.g., medication withdrawal, sleep deprivation).^18^, ^19^ In 1 study, reductions in episode starts and/or rates of long episodes in the first 1 to 2 weeks of initiation of an ASM was associated with efficacious ASMs (clinical efficacy at 3 months).^14^ To control for day‐to‐day variability, electrographic data before and after ASM initiation were binned in week intervals.^14^ In another study, the use of clinically effective ASMs was associated with a greater reduction in epileptiform activity than ineffective ASMs^15^ at 1 and 3 months after initiation of treatment. Similar to the previous study, there was variability in electrographic data, and the authors suggested that individual differences may contribute to the variability in electrographic activity. Additionally, the authors suggested that the value of RNS to detect a reduction in electrographic activity may depend on the specific electrographic variables selected, as variables might be informative for some, but not all, patients.^15^ Acquisition and analysis of RNS data across longer time frames to reduce variability and statistical error differentiates studies of RNS and daily ASMs with the present study that used a rescue (single‐dose) ASM. We chose to evaluate shorter time frames for RNS activity because a single dose of diazepam nasal spray has pharmacokinetic properties (time of onset and half‐life) that we hypothesized could be detected using shorter time intervals of electrocorticographic detections. The variability in the baseline RNS measures (number and duration of detections collected at 10‐min, hourly, and 24‐h intervals) may have masked any ASM effect. Although there were trends for reduced electrographic activity in 3 of the 4 participants, the reduction was not meaningful using the prespecified criteria (reduction of >1 SD), with the exception of the 24‐h interval for participant 5.

There is a need to identify new end points that could better characterize the efficacy of rescue medications.^12^ This small pilot study represented a first step to explore the potential of RNS to detect changes in clinical and subclinical electrographic events following the administration of a rescue medication. The reduction of seizures associated with acute repetitive seizure episodes seen in the clinical trials of diazepam as rescue medication was seen in studies with large sample sizes compared to the present study.^8^, ^9^, ^10^ The equivocal results presented here do not fully support or dismiss the viability of ambulatory electrographic activity as a marker of pharmacodynamic effect for rescue medication; as such, RNS might not be an ideal biomarker to demonstrate effectiveness for this type of treatment. The nature of intermittent use of rescue medication limits the durations of analyses as described in studies of daily ASM use, which were critical to control variability and detect statistical effects. In the present study, the 24‐h time interval appeared to be the most stable while also showing an apparent effect for at least 1 participant. Thus, cycles of epileptiform activity^20^ may potentially require further investigation and consideration as they may confound short‐term electrographic results. We attempted to control for circadian cycling by comparing 10‐min and 1‐h intervals at the same time of day pre‐ and post‐dose, but even using this strategy the variability among days in the baseline period was exceptionally high. Conducting the single‐dose trial multiple times (weeks to months) might be a way to control errors associated with cycles of epileptiform activity.

RNS detections are typically runs of inter‐ictal spikes or other patterns associated with seizure onset; however, the majority of detections are not associated with clinical seizures.^13^ Therefore, these findings should not be interpreted as providing information about the effectiveness of benzodiazepine therapy in terminating seizures. RNS detections were used for this study because of the high frequency, typically in hundreds per day, and could be more sensitive to the rapid onset of diazepam nasal spray. Long episodes as measured by the RNS system are more closely correlated with clinical seizures,^13^ but in this population, long episodes were too infrequent to measure a medication effect with onset over minutes–hours.

This pilot study had limitations. The magnitude of potential treatment effects was not robust relative to the high levels of variability in electrographic data. The sample size was small, participant recruitment was limited to those with RNS and confined to a single center, and electrocorticographic alpha and gamma spectral power were not measured. Moreover, we were unable to sufficiently control for cycles of epileptiform activity in individual participants. Abnormal electrographic activity can occur during sleep or with sleep deprivation,^12^ and 1 of the 4 participants fell asleep during the observation period (1 participant was excluded from the study because of seizures during baseline and observation periods). Participants' overnight sleep was not assessed, and given that this was a short‐term study in people with drug‐resistant epilepsy, acute disruptions (sleep, seizure) could substantially confound electrographic measurements and conclusions. Participants in this study had a mix of temporal lobe and frontal lobe seizure onset and there was no obvious trend seen. A larger sample size of different seizure localization subtypes could provide additional information.

## 5 CONCLUSION

Electrographic detections and durations of detections as recorded by RNS were not sensitive measures of short‐term (minutes–hours) pharmacodynamic effects of diazepam nasal spray in this group of participants. Large variations in day‐to‐day detection rates at identical times of the day plus variable durations of cyclic (e.g., daily, multiday) influences on detections may have masked a measurable drug effect.

## AUTHOR CONTRIBUTIONS

All authors contributed to conception, design, and development of the article, and all authors revised the manuscript critically for important intellectual content. All authors read and approved the final version of this manuscript for submission to *Epilepsia*.

## FUNDING INFORMATION

This study was funded by Neurelis, Inc. (San Diego, CA).

## CONFLICT OF INTEREST STATEMENT

**Dr Privitera** has served as a consultant and speaker for Neurelis, Inc.; Jazz Pharmaceuticals; and SK Life Science, Inc., He has received research support from the Epilepsy Foundation; GW Pharmaceuticals; Neurelis, Inc.; SK Life Science, Inc.; and Xenon. **Ms Mendoza** has nothing to disclose. **Dr Carrazana** is an employee of and received stock and stock options from Neurelis, Inc. **Dr Rabinowicz** is an employee of and has received stock options from Neurelis, Inc.

## ETHICAL STATEMENT

We confirm that we have read the Journal's position on issues involved in ethical publication and affirm that this report is consistent with those guidelines.

## IRB STATEMENT

Before study initiation, the study protocol, informed consent form, and other relevant study documentation were approved by ethics committees or institutional review boards at each site.

## Data Availability

All relevant data are within the paper.
